# ILC2 Cells Promote Th2 Cell Differentiation in AECOPD Through Activated Notch-GATA3 Signaling Pathway

**DOI:** 10.3389/fimmu.2021.685400

**Published:** 2021-06-18

**Authors:** Min Jiang, Ren Cai, Jing Wang, Zheng Li, Dan Xu, Jing Jing, Fengbo Zhang, Fengsen Li, Jianbing Ding

**Affiliations:** ^1^ Xinjiang Laboratory of Respiratory Disease Research, Traditional Chinese Medicine Hospital Affiliated to Xinjiang Medical University, Urumqi, China; ^2^ Clinical Medical Research Institute, First Affiliated Hospital of Xinjiang Medical University, Urumqi, China; ^3^ Department of Clinical Laboratory, First Affiliated Hospital of Xinjiang Medical University, Urumqi, China; ^4^ Department of Immunology, College of Basic Medicine, Xinjiang Medical University, Urumqi, China

**Keywords:** acute exacerbation of chronic obstructive pulmonary disease, Notch-GATA3 pathway, type 2 innate lymphoid cell, Th2 polarized, cell differentiation

## Abstract

This study is to investigate the capacity of type 2 innate lymphoid cells (ILC2s) in regulating the Th2 type adaptive immune response of acute exacerbation of chronic obstructive pulmonary disease (AECOPD). The study enrolled healthy people, stable chronic obstructive pulmonary disease (COPD) patients, and AECOPD patients. Flow cytometry was used to detect Th2 and ILC2 cells in the peripheral blood. In addition, ILC2s from the peripheral blood of AECOPD patients were stimulated with PBS, IL-33, Jagged1, DAPT, IL-33+Jagged1, IL-33+DAPT, and IL-33+Jagged-1+DAP *in vitro*. The levels of cytokines in the culture supernatant were detected by ELISA and the culture supernatant was used to culture CD4 ^+^ T cells. The mRNA and protein levels of Notch1, hes1, GATA3, RORα, and NF-κB of ILC2s were detected by real-time PCR and Western blot. The proportion of Th2 and ILC2s was significantly increased in the peripheral blood of AECOPD patients, alone with the increased *Notch1, hes1*, and *GATA3* mRNA levels. *In vitro* results showed that the mRNA and protein levels of Notch1, hes1, GATA3 and NF-κB were significantly increased after stimulation with Notch agonist, meanwhile, the level of type 2 cytokines were increased in the supernatant of cells stimulated with Notch agonist, and significantly promoted differentiation of Th2 cells *in vitro.* Disruption of Notch pathway weakened GATA3 expression and cytokine production, and ultimately affected the differentiation of Th2 cells. In conclusion, our results suggest that ILC2s can promote Th2 cell differentiation in AECOPD *via* activated Notch-GATA3 signal pathway.

## Introduction

Acute exacerbations of chronic obstructive pulmonary disease (AECOPD) is characterized by sudden deterioration of respiratory symptoms, rapid deterioration of respiratory function and poor prognosis (1, 2), which causes serious health and socioeconomic burden (3–6). AECOPD are the poor outcomes of COPD, leading to a decline in the quality of life of patients and an increase in mortality (7, 8). Studies have shown that the development of COPD is related to the T lymphocyte mediated inflammatory immune response and immune imbalance (9–12). Our previously results indicates that the differentiation of CD4 T cells into Th2 cells is crucial for the development of AECOPD (13). Several prior reports provide evidence suggesting that type 2 innate lymphoid cells (ILC2s) activity is essential for the efficient differentiation of Th2 cells (14–16). Our group found that ILC2s can promote the differentiation of Th2 cells in AECOPD through the direct action of MHCII (13). However, blocking MHCII molecules cannot completely reverse the differentiation effect of ILC2s on Th2. Therefore, we speculate that there may be other ways for ILC2s to promote Th2 differentiation.

Studies have found that in addition to expressing MHCII and costimulatory molecules (such as CD80, CD86, OX40L), ILC2 cells also participate in the efficient differentiation of Th2 cells *via* production of various cytokines (17, 18). ILC2 produce Th2 cytokines, such as IL-5, IL-13, and IL-4, which are essential for differentiation of CD4 T cells *in vitro* (19–23).Notch signaling is involved in the expression of these cytokines (24). Notch is a cell surface receptor, after activation by Jag1/Notch interaction, the intracellular portion of Notch is translocated to the nucleus and forms a complex with CSL protein (Notch/CSL). Notch/CSL binds to the 3′ of the *Il4* gene and directly regulates IL-4 expression (24). Notch signaling pathway promotes IL-4 by remodeling chromosomes through the synergistic effect of GATA3 expression (25). GATA3 is a key transcription factor that regulates the differentiation and function of ILC2 (26, 27). Considering the abovementioned points, we hypothesized that Notch-GATA3 pathway affects IL-5, IL-13, and IL-4 through ILC2s and regulates Th2 hyperactivity during AECOPD attacks.

In this study, to address possible roles of ILC2 cells in regulating AECOPD adaptive Th2 response *via* Notch-GATA3 pathway, we performed *in vitro* experiments using isolated ILC2s and CD4^+^ T cells. Our findings indicate that Notch-GATA3 pathway might play an important role in regulating the ILC2-mediated Th2 immunoresponse in AECOPD.

## Materials and Methods

### Patients

In this study, we included outpatients and inpatients in Xinjiang Uygur Autonomous Region Chinese Medicine Hospital from March 2019 to December 2019. All patients were diagnosed according to the diagnostic criteria for COPD. Among them, 50 patients (34 males and 16 females) were with AECOPD (infrequent exacerbator phenotype), with average age of 68.4 ± 9.1 years; and 50 patients (32 males and 18 females) were with stable COPD (infrequent exacerbator phenotype), with average age of 69.7 ± 7.5 years. COPD patients were treated with bronchodilators. AECOPD patients in the acute exacerbation period were treated according to the guidelines (https://goldcopd.org/). We also enrolled 50 healthy controls (HCs) (36 males and 14 females) with an average age of 66.6 ± 7.6 years. The clinical and demographic information of subjects were listed in **Table 1**. This study was approved by the Ethics Committee of Xinjiang Uygur Autonomous Region Chinese Medicine Hospital and written informed consent was obtained from each patient.

**Table 1:** Basic demographic characteristics and measurements of the study cohort.

Items	Health control (n = 50)	Stable COPD (n = 50)	AECOPD (n = 50)	Statistics	*P* value
Age, mean (SEM)	66.6 (7.6)	69.7 (7.5)	68.4 (9.1)	F (0.46)	0.63
Sex, n (%)					
Male	36 (72)	32 (64)	34 (68)	χ^2^ (0.74)	0.69
Female	14 (28)	18 (36)	16 (32)		
BMI, mean (SEM)	24.4 (3.8)	24.3 (3.4)	24.8 (3.3)	F (0.22)	0.8
Disease duration, mean (SEM)	——	14.8 (12.9)	14.1 (12.4)	*t* (0.52*)*	0.6
History of smoking, n (%)					
Yes	34 (68)	32 (64)	43 (86)	χ^2^ (6.91)	0.03
No	16 (32)	18 (36)	7 (14)		
Measurements, mean (SEM)					
FEV1/FVC	79.6 (5.4)	73.4 (11.8)	64.6 (24.4)	F(4.01)	0.02
FEV1%	92.1 (11.9)	70.4 (19.1)	59.7 (20.2)	F (14.9)	<0.0001
FVC%	115.9 (14.6)	90.7 (21.8)	80.5 (19.4)	F(11.2)	<0.0001
6MWD (m)	437 (85)	416 (96)	399 (92)	F (0.34)	0.72
CAT	10.6 (6.5)	10 (5.9)	11.7 (6.3)	F (0.06)	0.94
mMRC	1.3 (0.4)	1.6 (0.7)	1.8 (0.6)	F (1.87)	0.16

AECOPD, acute exacerbations of chronic obstructive pulmonary disease; BMI, body mass index; COPD, chronic obstructive pulmonary disease; FEV1, forced expiratory volume in one second; FVC, forced vital capacity; FEV1/FVC were determined post bronchodilator; HCs, health controls; 6MWD, 6-min walk distance; CAT, Assessment Test Scale; mMRC, the Modified Medical Research Council Dyspnea Scale. Data were presented by mean ± SEM or percentage. The comparison among the three groups was determined by one-way ANOVA. The comparison between two groups was determined by t test, or Chi-square test. A P<0.05 was considered statically significant.

### Diagnosis, Inclusion, and Exclusion Criteria

COPD and AECOPD diagnostic criteria were in line with the Global Strategy for the Diagnosis, Management, and Prevention of Chronic Obstructive Pulmonary Disease (2019 Revision), published jointly by the American Thoracic Society and the European Respiratory Society (28). The inclusion and exclusion criteria for COPD and AECOPD were as previously described (13). In detail, the inclusion criteria for COPD were as follows: 1) The Forced Expiratory Volume in One Second (FEV1) < 80% predicted and FEV1/forced vital capacity (FVC) < 70% after using bronchodilator; 2) no acute exacerbation; 3) no change in therapeutic schedule. Meanwhile, the inclusion criteria for AECOPD were as follows: COPD patients had cough in a short term and the cough was aggravated accompanied by purulent sputum, asthma, dyspnea, and fever. The exclusion criteria included the following: 1) patients with other respiratory diseases or other lung diseases with restrictive ventilatory impairment; 2) patients with severe systemic illnesses such as severe cardiovascular diseases, hepatorenal diseases, and hematopoietic system diseases; 3) patients who received antibiotics, hormones (such as glucocorticoids), or immunosuppressants in the previous 30 days before enrollment. All the HCs were of neither symptom of infection nor abnormal lung function.

### Lung Function, Physical Performance, and COPD-Related Questionnaires

Pulmonary function was evaluated using the Masterscreen PET 601-1/IP20 Respiratory Function Instrument (Jager, Germany). A 6-min walk distance (6MWD) test was administered in a 50-m indoor corridor by an expert investigator and was performed in accordance with the standards of the test. COPD assessment test (CAT), and the modified Medical Research Council scale (mMRC) were completed (29).

### Sampling

The peripheral blood was collected before antibiotic or hormone therapy. Peripheral blood mononuclear cells (PBMCs) were isolated from the whole blood by Ficoll density gradient method (30). Briefly, the samples were diluted with PBS at 1:1 ratio, mixed with equal volume of Ficoll-Hypaque Solution (Solarbio, Beijing, China), and centrifuged at 2000 r/min for 22 min at room temperature. The white layer was PBMCs. PBMCs were rinsed twice with PBS and then centrifuged at 1,500 rpm/min for 5 min at room temperature to collect the cell pellets. In order to obtain sufficient cells for real-time PCR, ELISA, and Western blot analysis, we pooled the peripheral blood of every five patients and obtained 10 samples in total.

### Flow Cytometry

Th2 surface staining was performed as previously described (31). Briefly, 100 μl of whole blood was incubated with FITC-CD4 (SK3, BD, San Jose, CA, USA), PE-Cy5-CD183 (1C6/CXCR3, BD, San Jose, CA, USA), and PE-CD196 (11A9, BD, San Jose, CA, USA) at room temperature for 30 min in the dark. Isotype control was added to block the nonspecific binding. The cells were lysed using red blood cell lysate for 10 to 15 min and then washed with PBS twice. All samples were detected by BD flow cytometry (LSR II, BD, USA), and analyzed using Kaluza software (Beckman Coulter, Inc) or FlowJo_V10 (Tristar, USA).

ILC2 staining: 200 μl of whole blood was incubated with the following antibodies (all from BD unless specified otherwise), including Lineage-FITC, CD3 (UCHT1), CD19 (HIB19), CD123 (7G3), CD11b (M1/70), CD11c (B-ly6), CD8 (RPA-T8), FceRI (AER-37 (CRA-1), CD14 (M5E2), CD4 (RPA-T4), CD56 (B159), CD45-APC-Cy7 (2D1), CRTH2-PerCP-Cy5.5 (BM16), CD127-PE-Cy7 (HIL-7R-M21), at room temperature for 30 min in the dark. Isotype control was added to block the nonspecific binding. The cells were lysed using red blood cell lysate for 10 to 15 min and then washed with PBS twice. All samples were detected by BD flow cytometry (LSR II, BD, USA), and analyzed using Kaluza software (Beckman Coulter, Inc) or FlowJo_V10 (Tristar, USA).

### Sorting and Treatment of ILC2s

Peripheral blood samples of the HC group, stable COPD group, and AECOPD group were collected. ILC2s from each group were then sorted by magnetic beads (STEMCELL, Vancouver, BC, Canada). Briefly, CRTH2^+^ cells were isolated by column-free immunomagnetic positive selection using antibody complexes and EasySep™ Releasable RapidSpheres™ (STEMCELL, Vancouver, BC, Canada). Then, bound magnetic particles were removed from the EasySep™-isolated CRTH2^+^ cells. The unwanted non-ILC2s were targeted for depletion using antibody complexes and EasySep™ Dextran RapidSpheres™ (STEMCELL, Vancouver, BC, Canada). The final isolated fraction contained highly purified ILC2s. The purity of the sorted ILC2 cells, which was analyzed by flow cytometry, was up to 95%. Then the sorted ILC2s were cultured *in vitro* as previously described (13). Briefly, the cells were seeded at 500 cells/well in 96-well plates and cultured with incomplete IMDM medium supplemented with 500 ng/ml IL-2 (200-02-10, PeproTech, Rocky Hill, NJ, USA), 500 ng/ml IL-25 (8134-IL-025, R&D, Minneapolis, MN, USA), 500 ng/ml IL-33 (R&D, Minneapolis, MN, USA), and 500 ng/ml TSLP (300-62-10, PeproTech, Rocky Hill, NJ, USA) for 5 days. Before use, ILC2s were washed once to remove residual IL-2, IL-25, IL-33 and TSLP. ILC2 cells were then divided into PBS group, IL-33 stimulation group (10 ng/ml, 10368-HNAE, Sino Biological Inc), Jagged 1 (Notch agonist) treatment group (10 ng/ml, 1277-JG-050, R&D Systems), DAPT (Notch inhibitor) treatment group (20 μM, D5942-5MG, SIGMA), IL-33+Jagged 1 treatment group, IL-33+DAPT treatment group, and IL-33+Jagged 1+DAPT treatment group. The cells were incubated at 37°C, 5% CO_2_ for 4 days. After that, cells and supernatant were collected for further analysis.

### CD4^+^ T Cell Isolation and Culture

CD4^+^ T cells were isolated from peripheral blood of the HCs with magnetic beads (Miltenyi Biotec, Bergisch Gladbach, Germany). Briefly, non-CD4^+^ cells, such as CD8^+^T cells, monocytes, neutrophils, eosinophils, B cells, dendritic cells, NK cells, granulocytes, γ/δ T cells, or erythroid cells were labeled by using a cocktail of biotin-conjugated antibodies. The cocktail contained antibodies against CD8, CD14, CD15, CD16, CD19, CD36, CD56, CD123, TCR γ/δ, and CD235a (glycophorin A). Subsequently, non-target cells were magnetically labeled with the CD4^+^T cell MicroBead Cocktail. Isolation of highly pure T cells was achieved by depletion of magnetically labeled cells. Flow cytometry detected the purity of isolated CD4^+^ cells, which was up to 95%. CD4^+^ T cells were cultured with plate-bound anti-CD3 (2 μg/ml) and soluble anti-CD28 (1 μg/ml) as well as with supernatants from ILC2s treated with PBS, IL-33, Jagged 1, DAPT, IL-33+Jagged 1, IL-33+DAPT, and IL-33+Jagged 1+DAPT in a 96-well plate at 2 × 10^4^ cells/well at 37°C, 5% CO_2_ for 2 days. After that, Th2 cells were detected by flow cytometry.

### Real-Time PCR

Total RNAs were extracted from ILC2s using Trizol LS (Thermo Fisher Scientific, Inc.). Then, the cDNA was reverse-transcribed from the isolated total RNA using Transcriptor first strand cDNA synthesis kit (Takara, Tokyo, Japan). Real-time PCR was performed with Power SYBR™ Green PCR Master (Thermo Fisher Scientific, Inc.) on a BIO-RAD CFX96 detection system (LSR II, BD, USA) according to the manufacturer’s instructions. Primers for these genes were listed in **Table 2**. The PCR system was as follows: 95°C for 5 min, followed by 40 cycles of 95°C for 30 s, and 60°C for 30s. The relative quantity of the target gene was calculated with 2^−△△Ct^ method and normalized to β-actin.

**Table 2:** Primers for RT-PCR.

Gene	Forward primer (5′→3′)	Reverse primer (5′→3′)	PCR product size (bp)	Tm (°C)
Notch	GAGGCGTGGCAGACTATGC	CTTGTACTCCGTCAGCGTGA	140	60
NF-κB	CCCACGAGCTTGTAGGAAAGG	GGATTCCCAGGTTCTGGAAAC	96	60
GATA3	GTGCATGACTCACTGGAGGACTTC	CATGTGGCTGGAGTGGCTGAAG	114	60
RORα	ACACCTTGCACAGAATATATCTAAATC	ACTAGTTTAACACGAAGATT	200	60
Hes1	CCAAGTGTGCTGGGGAAGTA	CACCTCGGTATTAACGCCCT	96	60
β-Actin	CATGTACGTTGCTATCCAGC	CATGTACGTTGCTATCCAGC	138	60

### Enzyme-Linked Immunosorbent Assay (ELISA)

Concentration of cytokine in cell-culture supernatants were detected using double antibody sandwich ELISA kits (Hangzhou Lianke Biotechnology Co., Ltd, Hangzhou, China). Briefly, the ELISA plate was coated with capture antibody. After the samples were added, the plates were further incubated with a horseradish peroxidase-conjugated secondary monoclonal antibody. After washing with PBST three times, tetramethylbenzidine was added in the dark for color development. Absorbance was determined at 450 nM and a reference wavelength of 570 nM using spectrophotometer (Thermo Fisher Scientific, Waltham, MA, USA). Concentrations were calculated based on a standard curve.

### Western Blot

ILC2 cells were lysed by cold RIPA buffer (87787, Thermo Fisher Scientific, Waltham, MA, USA). Total protein lysates (25 μg/lane) were separated *via* 12% SDS-PAGE and then transferred to polyvinylidene fluoride membranes. The membranes were subsequently blocked with 5% nonfat milk in Tris-Buffered Saline and Tween-20 (TBST, maintaining 20 mM Tris-HCl, 0.15 M NaCl, 0.05% Tween-20, pH 7.5) for 2 h at room temperature and further incubated overnight with rabbit anti-Hes1 (1:500, Abcam, Cambridge, MA, USA), rabbit anti-GATA3 (1:500, Abcam, Cambridge, MA, USA), rabbit anti-NF-κB (1:500), rabbit anti- Notch (1:500, Abcam, Cambridge, MA, USA), rabbit anti- RORα (1:1000, Abcam, Cambridge, MA, USA), and mouse anti-β-actin antibodies (1:800, Abcam, Cambridge, MA, USA) at 4°C according to the manufacturer’s instructions. After washing with Tris-Buffered Saline and Tween 20 (TBST, Beyotime Biotech. Shanghai, China) for three times, the membranes were incubated with the secondary antibody (Abcam, Cambridge, MA, USA) and finally processed using an enhanced chemiluminescence (ECL) reaction kit (Cell Signaling Technology, Danvers, MA, USA). The relative band densities of the target proteins relative to the β-actin band density were quantified using the Bio-Rad Quantity One 1-D analysis software package (Bio-Rad, Hercules, CA, USA).

### Statistical Analysis

Statistical analysis was performed using Graphpad Prism 5 (Graphpad Software Inc., San Diego, CA, USA). All data are expressed as mean ± SEM. One-way ANOVA was used to compare the data among multiple groups followed by LSD-t test. Count data were expressed as percentages and analyzed by χ ^2^ test. A P value < 0.05 indicates statistical difference.

## Results

### Clinical Data of Patients

A summary of patient demographics is shown in **Table 1**. No statistically significant difference was observed in clinical data of patients at baseline among the groups. However, the FEV1/FVC of AECOPD group was obviously lower than that in HCs (all *P* < 0.001). In addition, FEV1% and FVC% of stable COPD and AECOPD patients were also obviously lower than in the HCs groups (*P* < 0.01).

### Th2 Polarization and ILC2 Increase in AECOPD Patients

**Figure 1:**
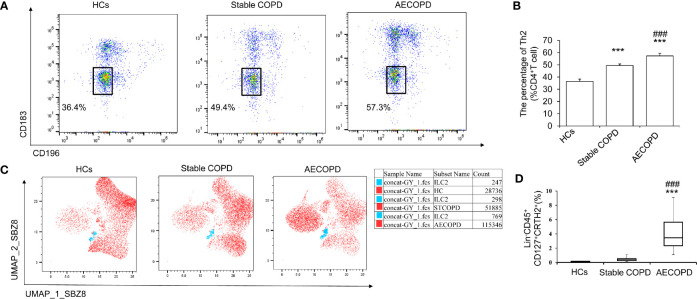
Th2 and ILC2 cells in different groups. Th2 cells and ILC2 cells in healthy control group, stable COPD group and AECOPD group were analyzed by flow cytometry. **(A)**Th2 (CD183^−^ CD196^−^) was labeled with specific antibodies and quantified in each groups. **(B)** Percentage of Th2 cells. **(C)** Uniform manifold approximation and projection (UMAP) analysis of ILC2 cells in peripheral blood among the three groups. **(D)** Percentage of ILC2s in three groups. Data are shown as mean ± SEM. n=50. ^***^ *P* < 0 001, compared with the control group. ^###^ *P* < 0.001 compared with the stable COPD group.

To observe the connection between Th2 cell polarization and ILC2 in AECOPD patients, Flow cytometry was used to detect the proportion of Th2 and ILC2 cells in peripheral blood of AECOPD group, stable COPD group, and HCs group. There was more Th2 cell in AECOPD patients (57.3% ± 0.25%) (*P* < 0.05, **Figures 1A, B**). The percentage of ILC2s in the peripheral blood of the AECOPD group was (4.08% ± 2.23%), which was significantly higher than that in the stable COPD group (0.45% ± 0.25%) and in the healthy group (0.14% ± 0.04%) (all *P* < 0.001, **Figures 1C, D** **)**. These results suggest that increased ILC2 cells may play a potential role in the initiation, amplification, and modulation of type 2 immune responses in the pathogenesis of AECOPD.

### Expression of *Notch, Hes1, GATA3*, and *RORα* Genes in ILC2s of AECOPD Patients

**Figure 2:**
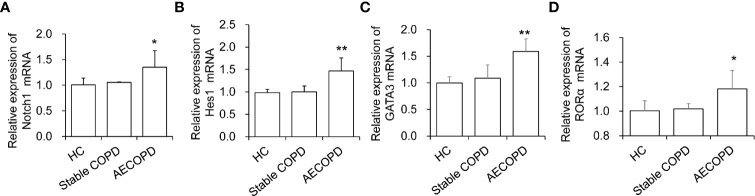
Levels of ILC2 related transcription factors in different groups. The mRNA levels of **(A)** *Notch1*, **(B)** *Hes1*, **(C)** *GATA3* and **(D)** *RORα* were tested by real-time PCR. Data were normalized to control group and represented as mean ± SEM. n=50. ^*^ *P* < 0.05, ^**^ *P* < 0.01 compared with control group.

We next assessed Notch pathway and key factors that are known to play roles in ILC2 development and function. First, we isolated ILC2 cells from the peripheral blood of patients in the HC group, COPD group and AECOPD group. The purity of the sorted ILC2 cells was up to 95%. Then the expression of Notch, Hes1 genes in ILC2s were performed qPCR analyses. The results showed that in AECOPD patients, *Notch* mRNA level increased 1.34 folds than HCs and *Hes1* mRNA level increased 1.5 fold than HCs group (*P* < 0.05, and *P* < 0.01, respectively, **Figures 2A, B** **)**. The factor RORα is essential for differentiation of ILC2 cells. In addition, ILC2s depend on GATA3 for development and function. As expected, we found that GATA3 and RORα mRNA in the AECOPD group were also significantly higher than those in the HC group, with the increase of 1.59 fold and 1.18 fold, respectively (all *P* < 0.05, **Figures 2C, D** **)**. The above data indicate that Notch pathway might involve in the development and function of ILC2s.

### ILC2 Are Activated by Notch Pathway

**Figure 3:**
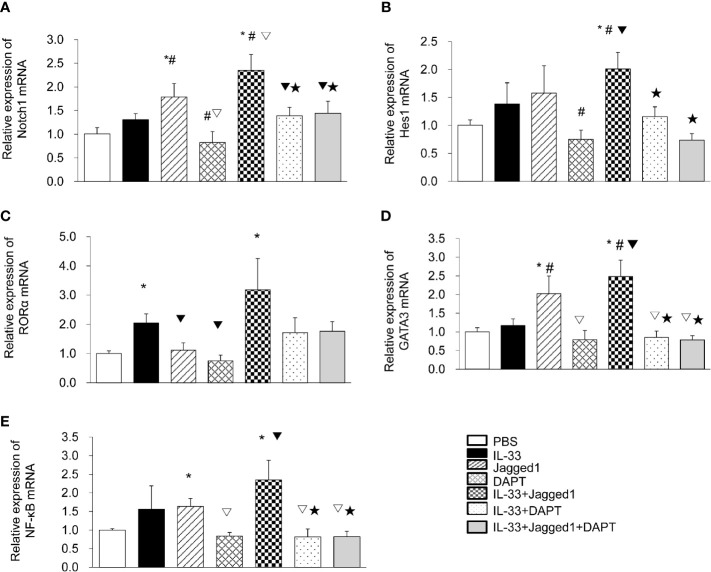
The mRNA levels of *Notch1*, *Hes1*, *RORα*, *GATA3* and *NF-κB* in different groups. The relative levels of *Notch1* **(A)**, *Hes1* **(B)**, *RORα* **(C)**, *GATA3* **(D)**, and *NF-κB* **(E)** mRNA in the supernatant of PBS group, IL-33 stimulation group, Jagged 1 treatment group, DAPT treatment group, IL-33 + Jagged 1 treatment group, IL-33 + DAPT treatment group were determined by real-time PCR. Data are shown as mean ± SEM. n=10. ^*^ *P* < 0.05 compared with PBS group, ^#^P < 0.05, compared with IL-33 group, ^▽^ *P* < 0.05 compared with Jagged1 group, ^▼^ *P* < 0.05 compared with DATP group, ^★^ *P* < 0.05 compared with IL-33+Jagged1 group.

To determine the role of the Notch signaling pathway in ILC2 cells, we isolated ILC2 cells (95% purity) from the peripheral blood of patients in the AECOPD group and further treated them with IL-33, Jagged1, and DATP alone or in combination. Real-time PCR results showed that the *Notch* mRNA levels in Jagged 1 group and IL-33 + Jagged 1 group were significantly higher than PBS group, IL-33 group, DAPT group, IL-33 + DAPT group, and IL-33 + Jagged 1+ DAPT group (all *P*<0.05, **Figure 3A**). *Hes1* mRNA levels in IL-33 + Jagged 1 group was significantly higher than DAPT group, IL-33 + DAPT group and IL-33 + Jagged 1+ DAPT group (all *P*<0.05, **Figure 3B**). Meanwhile, *RORα* mRNA in IL-33 and IL-33 + Jagged 1 groups were also significantly higher than other groups (all *P*<0.05, **Figure 3C**). As a consequence, *GATA 3* mRNA in Jagged 1 group and IL-33 + Jagged 1 group were significantly higher than other groups (P <0.05) (**Figure 3D**). Besides, in the Jagged 1 group and IL-33 + Jagged 1 group, *NF-κB* mRNA levels increased when compared with that in the PBS group (all *P <*0.05). However, in DAPT and IL-33 + DAPT group, *NF-κB* mRNA decreased compared with that in the IL-33 + Jagged 1 group (P<0.05, **Figure 3E**).

**Figure 4:**
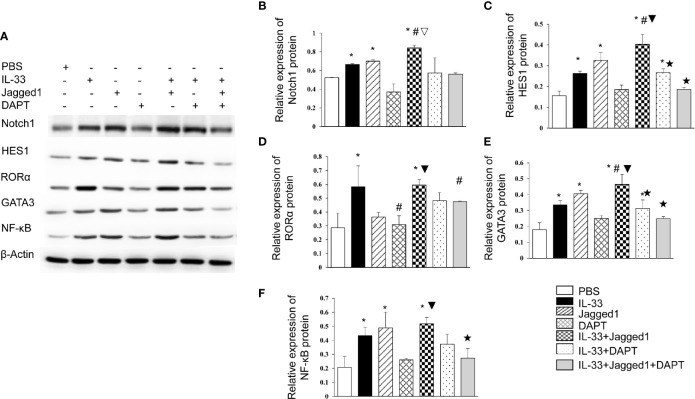
Protein levels of Notch1, Hes1, RORα, GATA3 and NF-κB in different groups. The protein expressions of Notch1, Hes1, RORα, GATA3 and NF-κB in the supernatant of PBS group, IL-33 stimulation group, Jagged 1 treatment group, DAPT treatment group, IL-33 + Jagged 1 treatment group, IL-33 + DAPT treatment group were determined by Western Blot. **(A)** Representative Western blot results of RORα, Notch1, Hes1, GATA3 and NF-κB in each treatment group. Expression of **(B)** Notch1, **(C)** Hes1, **(D)** RORα, **(E)** GATA3, and **(F)** NF-κB was measured by Western Blot relative to β-actin. Data are shown as mean ± SEM. n=10. ^*^P < 0.05 compared with PBS group, ^#^ *P* < 0.05, compared with IL-33 group, ^▽^ *P* < 0.05 compared with Jagged1 group, ^▼^ *P* < 0.05 compared with DATP group, ^★^ *P* < 0.05 compared with IL-33+Jagged1 group.

We also got the similar results by detection of protein levels for Notch, HES1, RORα, GATA3, and NF-*κ*B (**Figure 4**). In detail, Notch1 protein expression in IL-33 group, Jagged 1 group and IL-33 + Jagged 1 group was significantly higher than that in PBS, DAPT, IL-33 + DAPT and IL-33 + Jagged 1+ DAPT groups (all *P*<0.05, **Figures 4A, B** **)**. HES1 protein expression increased in IL-33 group, Jagged 1 group and IL-33 + Jagged 1 group. However, its level in DAPT, IL-33 + DAPT group and IL-33 + Jagged 1+ DAPT groups was significantly lower than that in IL-33 + Jagged 1 group (all *P*<0.05, **Figures 4A, C**). RORα protein was elevated in IL-33group and IL-33 + Jagged 1 group (all *P*<0.05, **Figures 4A, D** **)**. The change trend of GATA3 and NF-κB proteins in each group was consistent to that of HES1. Their levels were increased in IL-33 group, Jagged 1 group and IL-33 + Jagged 1 group. However, their levels in DAPT, IL-33 + DAPT group and IL-33 + Jagged 1+ DAPT groups were significantly lower than those in IL-33 + Jagged 1 group (all *P*<0.05, **Figures 4A, E, F**). Therefore, we believed that Notch agonists promoted the expression of GATA3, and thus promoted the expression of NF-κB. When the DAPT was added, the mRNA and protein expression of HES1, GATA3, and NF-κB were inhibited.

These results suggest that the activation of Notch-GATA3 pathway may promote the development and function of ILC2 cells.

### Notch Pathway Is Involved in Cytokine Production of ILC2 Cells

**Figure 5:**
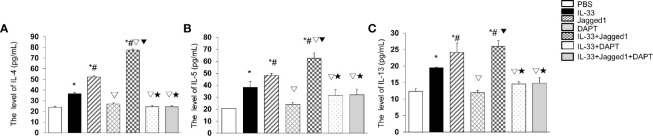
Cytokines after IL-33, Jagged 1 and DATP treatment. Level of **(A)** IL-4, **(B)** IL-5 and **(C)** IL-13 in the supernatant of PBS group, IL-33 stimulation group, Jagged 1 treatment group, DAPT treatment group, IL-33 + Jagged 1 treatment group, IL-33 + DAPT treatment group were detected by ELISA. Data are shown as mean ± SEM. n = 10. ^*^ *P* < 0.05 compared with PBS group, ^#^ *P* < 0.05 compared with IL-33 group, ^▽^ *P* < 0.05 compared with Jagged1 group, ^▼^ *P* < 0.05 compared with DATP group, ^★^ *P* < 0.05 compared with IL-33+Jagged1 group.

To investigate whether Notch activation promotes the cytokine production by ILC2 cells, the levels of IL-4, IL-5, and IL-13 in the culture supernatant were detected by ELISA. The results showed a significant increase of IL-4 in Jagged1 (52.27 ± 2.67 pg/ml) and IL-33 + Jagged1 groups (77.44 ± 10.00 pg/ml) when compared to PBS group (23.78 ± 1.91 pg/ml) (*P* < 0.05, **Figure 5A**). However, after DAPT treatment, IL-4 was significantly decreased (26.97 ± 1.98 pg/ml) (*P* < 0.05, **Figure 5A**), IL-33 + DAPT groups (24.44 ± 1.81 pg/ml) (P <0.05, **Figure 5A**), and even in IL-33 + Jagged 1+ DAPT group (24.41 ± 1.22 pg/ml) (P <0.05, **Figure 5A**). Meanwhile, the levels of IL-5 and IL-13 showed a similar trend to IL-4. In detail, there was a significant increase of IL-5 and IL-13 in Jagged1 (48.27 ± 1.64 pg/ml and 24.15 ± 2.80pg/ml) and IL-33 + Jagged1 groups (62.65 ± 4.27 pg/ml and 25.98 ± 1.72 pg/ml) compared to PBS group (20.70 ± 0.06 pg/ml and 12.34 ± 0.81 pg/ml) (*P* < 0.05, **Figures 5B, C**). But after DAPT, their levels were significantly decreased (20.08 ± 1.66 pg/ml and 11.94 ± 0.73 pg/ml) (*P* < 0.05), IL-33 + DAPT groups (31.05 ± 4.81 pg/ml and 14.61 ± 0.62 pg/ml) (P > 0.05, **Figures 5B, C**) and even in IL-33 + Jagged 1+ DAPT group (32.06 ± 4.29 pg/ml and 14.83 ± 1.63 pg/ml). There was no significant difference between IL-33 alone and IL-33+DAPT groups. These results demonstrate that Notch-GATA3 pathway could affect the secretion of IL-4, IL-5 and IL-13, and disruption of Notch-GATA3 pathway weakens the secretion of IL-4, IL-5, and IL-13.

### Notch-GATA3 Pathway Induce Th2 Polarization Capacity of ILC2 Cells

**Figure 6:**
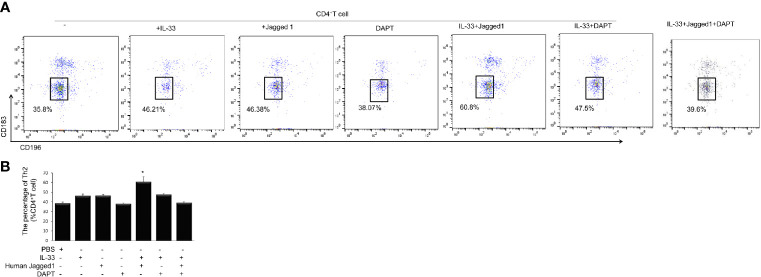
Th2 cells after treatment in different groups. Level of Th2 cells after treatment with the supernatant of PBS group, IL-33 stimulation group, Jagged 1 treatment group, DAPT treatment group, IL-33 + Jagged 1 treatment group, IL-33 + DAPT treatment group was determined by flow cytometry. **(A)** Th2 (CD183^−^ CD196^−^) was labeled with specific antibodies and quantified in each treatment groups. **(B)** Percentage of Th2 cells. Data are shown as mean ± SEM. n=10.^*^ *P* < 0.05 compared with CD4^+^ T cell group.

To investigate whether Notch-GATA3 pathway activates IL-4, IL-5, and IL-13 produced by ILC2 to promote Th2 polarization of CD4 T cells, an *in vitro* experiment was performed. CD4^+^ T cells were cultured with the supernatants from PBS control group, IL-33 group, Jagged 1 group, DAPT group, IL-33 + Jagged 1 group, IL-33 + DAPT group and IL-33 + Jagged 1+ DAPT group respectively. Flow cytometry showed that the supernatant of IL-33 + Jagged 1 group significantly promoted differentiation of Th2 cells *in vitro* compared to other groups (P < 0.05, **Figures 6A, B**). Taken together, these results suggest that ILC2 could induce the differentiation of CD4^+^ T cells into Th2 *via* the activation of Notch-GATA3 pathway.

## Discussion

Studies found that Th2 cells were dominated in AECOPD (9, 13, 32), in addition,Th2 immune responses are orchestrated by several cell types(such as ILC2, DC, basophils and epithelial cells) (33). Studies demonstrated that ILC2s were involved in the pathogenesis of COPD (34–36). Silver et al. found that ILC2 cells were decreased in mice with AECOPD caused by viral infection (37). In this study, we investigated the possible interaction by which ILC2 cells regulate Th2 cell polarization in an indirect way. We found increased ILC2s in peripheral blood of AECOPD patients, which is inconsistent with the findings by Silver et al. (37). This may because ILC2s are heterogeneous and have different roles in different tissues and at different disease stages. Further studies are needed to verify these findings. In total, our data demonstrate that the ILC2s enhance effector functions of Th2-type CD4 T cells *via* activated Notch-GATA3 pathway *in vitro.*

Notch signaling pathway is a highly conserved pathway that can regulate the development of various species by influencing cell lineage decision and differentiation at all stages (38–40). In those studies, Notch signaling was critical for immune regulation through T cell activation and Th cell differentiation. In this study, we observed that mRNA levels of *Notch1* and *Hes1* were significantly increased in ILC2 cells of patients with AECOPD, suggesting that Notch pathway can affect the function of ILC2 cells in patients with AECOPD.

In addition, previous studies have found that Notch signaling is an important transcription factor that triggers ILC2 plasticity (36, 41).On the other hand, the development of ILC2 is also regulated by the GATA-3 transcription factor. It is shown that Notch1 combined with the GATA3 promoter enhances the Notch pathway, which in turn increases GATA3 gene expression (42). In our study, we found that the ILC2 cell-related transcription factors Notch and GATA3 were increased significantly in AECOPD patients. *In vitro* experiment, we found that the mRNA levels of *Notch, Hes1, Gata3*, and *NF-κB* in the Notch agonist group were significantly higher than those in the PBS group. However, in the Notch blocker group, these mRNA levels decreased. Similarly, Western blot results showed that the protein levels of Notch, HES1, GATA3, and NF-κB in IL-33, Notch agonist group were significantly higher than those in PBS and DAPT group. However, in the Notch blocker group, these mRNA levels decreased. Our data demonstrate that the Notch signaling pathway may stimulate the activation of GATA3.

Studies have also shown that ILC2 can promote the differentiation of Th2 cells (43, 44) by secreting cytokines (17, 45). Here, we conducted *in vitro* cell culture and intervention experiments to check whether the Notch signaling pathway regulates GATA3 to promote type 2 cytokines secretion. In addition, IL-4, IL-5, and IL-13 secretion increased significantly in the Notch agonist group. After treating T cells with these culture supernatants, the differentiation ratio of Th2 cells increased significantly. Zhang (46) found that the Notch pathway could cause plasticity changes in ILC2 and drive the appearance of highly proinflammatory congenital lymphocytes. Furthermore, the activated Notch signaling pathway involved in the regulation of GATA3, which directly affects the expression of IL-4 gene through chromatin remodeling and epigenetic modification (47). Our results indicate that the Notch signaling pathway might promote the expression of Th2-related cytokines, thereby creating the environment necessary for the polarization of Th2 cells, and then transforming the adaptive immune response into Th2 type. However, our results also suggest that when IL-33 is present, DAPT may have off-target effects that could be decreasing IL-33–induced cytokines and thus decreasing Th2 cell differentiation. The underlying mechanisms still need further investigation. Together, these results indicate that the Notch-GATA3 pathway is involved in ILC2-mediated upregulation of Th2 *in vitro*. However, the potential underlying mechanism still needs further investigation.

In conclusion, our findings indicate that the Notch-GATA3 pathway influences Th2 cytokines secretion by ILC2s, thereby promoting Th2 cell polarization. Interruption of the Notch pathway could weaken ILC2 mediated Th2 polarization. This work indicates a novel mechanism for Th2 response in AECOPD patients and is a proof-of-concept study for targeting ILC2 in AECOPD patients as a novel intervention for advanced AECOPD. However, the underlying mechanism of how ILC2 regulates the differentiation of Th2 cells through Notch-GATA3 pathway in AECOPD remains unclear and needs to be further studied.

## Data Availability Statement

The raw data supporting the conclusions of this article will be made available by the authors, without undue reservation.

## Ethics Statement

The studies involving human participants were reviewed and approved by the Ethics Committee of Xinjiang Uygur Autonomous Region Chinese Medicine Hospital. The patients/participants provided their written informed consent to participate in this study.

## Author Contributions

JD and FL conceived and designed the experiments. MJ, RC, ZL, JW, DX, JJ, and FZ performed the experiments. MJ, RC, and JW analyzed the data. JD collected the fund. MJ, RC, and JD wrote the manuscript. JD and FL revised the manuscript. All authors contributed to the article and approved the submitted version.

## Funding

This work was supported by National Natural Science Foundation Regional Fund Project (81760793).

## Conflict of Interest

The authors declare that the research was conducted in the absence of any commercial or financial relationships that could be construed as a potential conflict of interest.
